# White blood cell classification using custom deep neural network and visualizing features of the images using heatmaps

**DOI:** 10.1038/s41598-026-35138-9

**Published:** 2026-03-18

**Authors:** Sahebgoud Hanamantray Karaddi, Hanumantharao Bitra, Sai Sambasiva Rao Bairaboina, Bharath Reddy Gudibandi

**Affiliations:** 1 Department of Electronics and Communication Engineering, Kishkinda University, Ballari, 583120 Karnataka India; 2 School of Electronics Engineering, VIT-AP University, Amaravathi, 522237 India; 3 Department of Computer Science and Engineering, Siddhartha Academy of Higher Education, Deemed to be University, Vijayawada, 520007 India

**Keywords:** Biomedical engineering, Electrical and electronic engineering, Engineering

## Abstract

Blood count is a key method for diagnosing diseases by analyzing blood cell images using advanced equipment. Traditionally, this process involves invasive techniques and is time-consuming and costly. Modern approaches leverage deep learning (DL) to streamline this process, making it faster and more cost-effective. Given the similarity among blood cell images, distinguishing various blood types by sight is challenging. To address this, we propose a Customized deep neural network (CDNN) to accurately classify different types of blood cells while avoiding overfitting and degradation. CDNN uses a unique DL architecture to classify white blood cells (WBCs). We validated this architecture using the Raabin WBC and BCCD datasets. The model is optimized through preprocessing techniques such as normalization and data augmentation. Simulations were conducted with a batch size of 64, utilizing the Adam optimizer over 50 epochs. Our model achieved a high accuracy of 97.97% on the Raabin dataset and 99.64% on the BCCD dataset, outperforming existing state-of-the-art models. To understand the model’s classification process, we applied G-CAM and LIME. These results suggest that CDNN can be developed into clinically useful solutions for detecting WBCs in blood cell images, significantly improving diagnostic efficiency.

## Introduction

Leukocytes, sometimes referred to as white blood cells(WBCs), are critical to the body’s defense against dangerous pathogens, such as bacteria and viruses, as well as external invaders. Basophil, Neutrophils, eosinophils, lymphocytes and monocytes are the other five primary subtypes of WBCs. They can also be identified by their morphological and functional characteristics^1^. Because the number of leukocyte subtypes is of great importance to the health care system, WBC counts are critical for assessing the presence and prognosis of disease. These counts are often performed manually, although they can be used in laboratories without access to automated equipment^2^. The blood sample is examined under a microscope by a pathologist using the manual differential method to count and categorise the WBCs^3^. Blood samples are often examined by automated equipment using Coulter counting, static and dynamic light scattering, and cytochemical techniques. These techniques involve the analysis and grouping of the data into defined categories that correspond to the various leukocyte types^4–6^. The manual differential approach is thought to be a preferable choice for assessing the quantity and categorization of these WBCs since automated findings may be unreliable in the presence of abnormal or aberrant WBCs.

WBCs of each kind have distinct defensive roles against alien organisms^7^. One of the major white blood cells that defends against germs is the neutrophil. Basophils are involved in allergic responses, whereas eosinophils kill parasites and have a function in allergies. Monocytes enter cells and obliterate the body’s damaged tissues. Complex cells called lymphocytes regulate cell-mediated immunity. Lymphocytes recall invading bacteria and viruses because they are entirely distinct from other WBCs. Eosinophils contribute to tissue injury and inflammation in numerous disorders. Additionally, they play a crucial function in combating viral infections.

WBC subtypes are often distinguished by microscopic examination of the blood smear and evaluation of the morphologic characteristics of the nucleus and cytoplasm. These methods can be labor- and time-intensive, and they heavily depend on the investigator’s expertise^8^. WBCs have also been analyzed using a completely automated blood cell analyzer. These devices can’t be extensively employed at the point-of-care or in urban hospitals since they frequently have large test sample requirements and are costly^9^. Because of this, researchers have created automated, quicker methods for leukocyte analysis utilising computer vision techniques^3,10–13^. Several methods for classifying and segmenting WBCs have been proposed in light of recent developments in machine learning and computer vision, ranging from more basic deep learning (DL) techniques^14–17^ to more established machine learning models like Support Vector Machine^18^ and Naive Bayesian^19^. Convolutional Neural Networks (CNNs) have demonstrated outstanding performance in the analysis of medical images when using techniques^20–26^. Despite the fact that computational methods offer a quicker, more affordable, and more repeatable way to classify WBCs, automation of the computational procedure to attain clinical standards of accuracy and reliability in WBC classification is still being developed. The main contribution of this work are

- We proposed custom deep neural network (CDNN) for the classification of WBCs.
- To avoid over-fitting K-fold cross validation is used with K=5.
- We used two datasets such as balanced dataset (BCCD dataset) and unbalanced dataset (Raabin Blood dataset) to check the working efficiency of the proposed architecture.
- We tuned our proposed architecture using hyper-parameters
- Used eight different DL-models for the classification and also compared with the proposed model.
- Applied the visualization techniques to study and distinguish the different classes.
- Miss-classification analysis is done.

## Related work

White Blood Cell (WBC) classification is a critical task in medical diagnosis as it helps in identifying the type and count of WBCs in a blood sample. models have shown promising results in this task. In this literature review, we will explore the recent advancements in WBC classification using models.

Many techniques have been presented in past few years for classifying WBCs. To differentiate the four and five kinds of WBCs, Diouf et al.^27^ suggested a seven-layer convolutional neural network employing conventional folding and max-pooling techniques. The classification task could be completed by the suggested model with 97% accuracy. A fresh segmentation technique was presented by Mu-ChunSu et al.^28^ to separate a WBC from the smear images. The suggested approach consists of WBC segmentation, feature extraction, and classifier creation. The WBC area on the HSI colour space is located via the segmentation method. The second stage involves retrieving three categories of features for categorization: geometrical, color, and local directional pattern (LDP) characteristics. Following that, three distinct neural network classifiers are employed, namely the multi-layer perceptron (MLP)^29^, support vector machine (SVM), and hyper rectangular composite neural networks (NN). These classifiers achieved accuracies of 99.1%, 97.5%, and 88.9% respectively. Ozyurt proposed a fused convolutional neural network (fCNN) model for white blood cell (WBC) detection. The model utilized MRMR feature selection and an extreme learning machine. This approach has a 96% accuracy rate in categorizing white blood cells (WBC). Vatathanavaro et al. (2018) investigated the categorization of five distinct types of white blood cells using two convolutional neural network architectures, namely VGG-16 and ResNet-50. The ResNet-50 classifier is the most precise, boasting an accuracy rate of 88.3%. In order to extract nucleus characteristics, Theera-Umpon^30^ used a brand-new collection of features created utilising mathematical morphology. Then, to identify the kind of WBC, these characteristics were utilised to train the artificial neural network and Bayes classifier. By modifying the five fold cross validation on pictures of blood cells, the scientists obtained encouraging and reliable results. A Generative adversarial network(GAN) for image enhancement and a cutting-edge deep neural network for categorising white blood cells were recently suggested by Almezhghwi et al.^31^. With a classification accuracy of 98.8%, the suggested approach can classify white blood cells. Using statistical pattern analysis, Madhumala et al.^32^ presented a systematic method to categorise WBCs. Based on the roundness, form factor, solidity, and compactness shape parameters, the WBCs are categorisedS using a Naive-Bayes technique. The overall accuracy achieved with this method is 83.2%. K-means clustering was suggested by Habibzadeh et al.^33^ as a technique for classifying and dividing WBCs, whereas SVM^34^ and NNs were utilised for classification. The authors classified WBCs with an accuracy of 80%. In order to recover WBCs, Liqun et al.^35^ increased classification accuracy based on feature weight K-means clustering. Neelam et al.^36^ recommended utilising K-Means Clustering first, then the Expectation Maximization algorithm, to divide and categorise WBCs. For categorization, they employed SVM and NN. They detected WBC with 80% success using this technique. In Nisha et al.^37^ identified WBC pictures in the HSV space using the S component. They detected WBC with a 94% success rate on average. In Arslan et al.^38^ suggested a technique based on the watershed transform algorithm in imaging of bone marrow and peripheral blood smear. WBCs were divided with 94% accuracy. In 2015, Prinyakupt^39^ suggested an approach that includes preprocessing, core segmentation, cell segmentation, feature extraction, feature selection, and classification. They performed the categorization of 4 cells and Basophils and Non-Basophils. With the confined data cell set, they attained a success rate of 98% to 99%. Using median filtering in grey-level images and histogram entropy classification thresholding, Sawsan et al.^40^ categorized five cell types made up of 70 images with 91% accuracy in their research from 2000.

In aforementioned model used pre-trained CNN and one dataset to classify the WBCs into five classes. In these, they have mentioned about prevention of degradation issue and they have not used cross validation to avoid the over-fitting. Also, due to more layers in the CNN architecture they face the gradient vanishing problem. The vanishing gradient problem arises when neural networks are trained using gradient-based learning techniques and back-propagation. The gradient in this issue decreases steadily during training, making it impossible to train neural networks since the network weights are fixed. So, to prevent this vanishing gradient problem we used residual blocks.

## Datasets

**Fig. 1:**
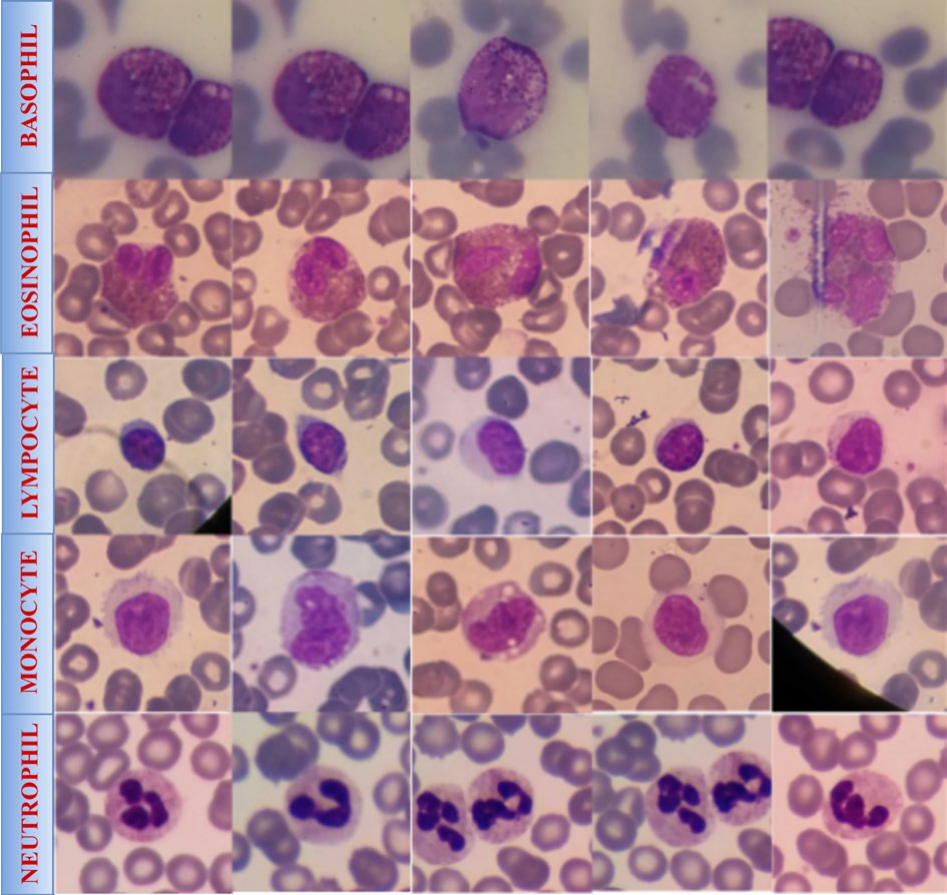
Example for WBCs datasets.

This section describes the datasets used for the classification WBCs. In this we have used two different datasets i. Raabin WBC^41^ ii. Blood cell images from Kaggle^42^. Raabin datasets consists of 8162 images that have five different classes Bosophil has 301 images, Eosinophil has 1066, Lymphocyte has 3000, Monocyte has 795, and Neutrophil has 3000 images. These all images are in .jpeg format. Second dataset consists of 6400 images that have four classes and uniformly distributed in four classes. In second dataset Eosinophil has 1600, Lymphocyte has 1600, Monocyte has 1600, and Neutrophil has 1600 images. The distribution of these datasets is shown in the Table 1 and sample images of each classes are represented in the Fig. 1.

The Raabin-WBC and BCCD datasets are frequently used for WBC classification tasks in medical imaging and machine learning research for several reasons: **Availability:** Both are publicly accessible datasets, making them valuable resources for researchers to develop and test WBC classification algorithms without the challenges of collecting and annotating large medical datasets from scratch. **WBC-specific focus:** Both datasets are specifically designed for tasks involving the identification and classification of WBCs in blood smear images, making them directly relevant to the problem. These two datasets are utilized in conjunction or one may be selected based on particular research objectives. Raabin-WBC is frequently favored for developing robust and generalized models because of its size and quality. BCCD is advantageous for assessing model performance under more demanding settings due to its noise and inferior image quality, occasionally utilizing data augmentation to enhance its size. The integration of these datasets for a thorough assessment of WBC categorization techniques under various data situations and scales.

**Table 1:** Dataset distribution of BCCD and Raabin WBCs.

Classes	Raabin WBCs	BCCD dataset
Basophil	301	—
Eosinophil	1066	1600
Lymphocyte	3000	1600
Monocyte	795	1600
Neutrophil	3000	1600
**Total**	**8162**	**6400**

All these images are resized to [227 227] and normalized all pixel to the range [0 1]. Then we have used DA to avoid imbalancing for Raabin WBC datasets and to avoid over-fitting issue for both dataset. In this, we used random rotation with value[-50 50],random reflection value 1, and random shear value [-5 5].

## Method

**Fig. 2:**
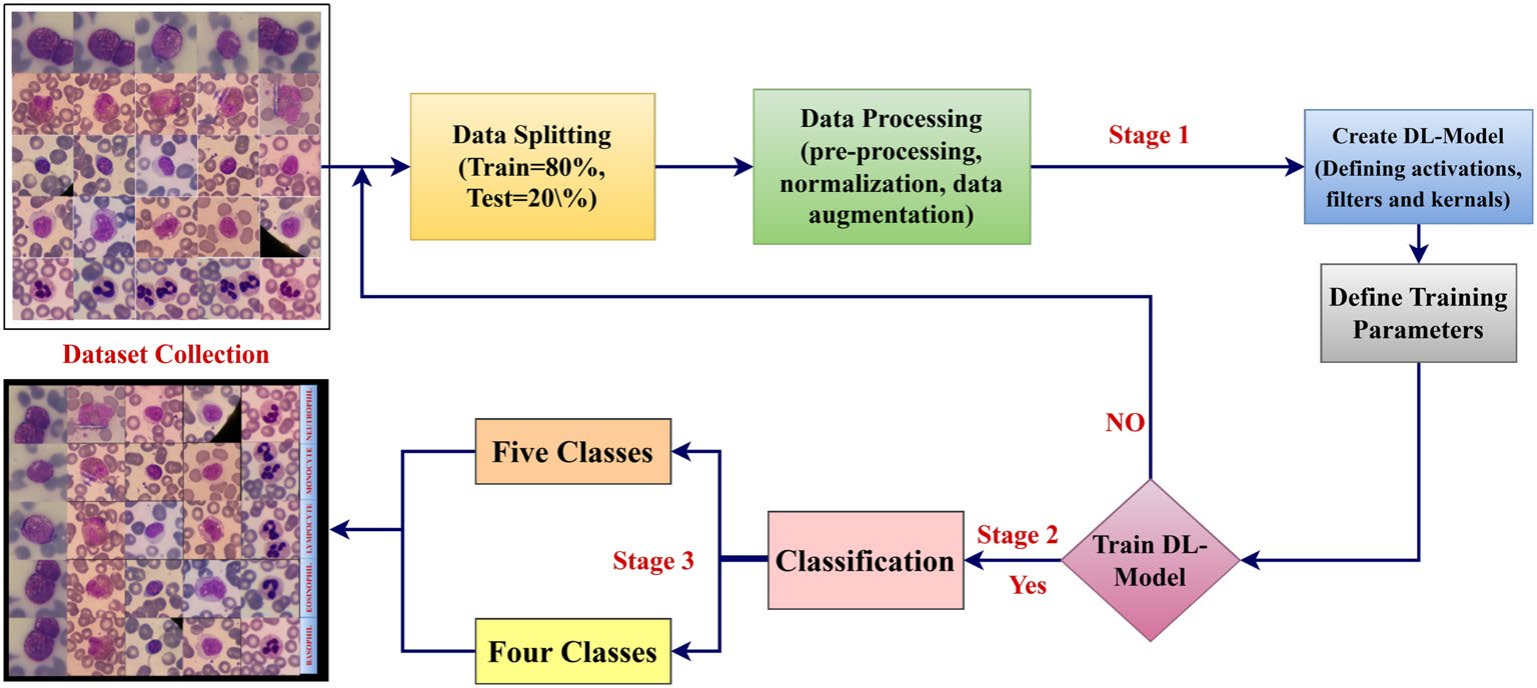
Classification of WBCs using CDNN.

**Fig. 3:**
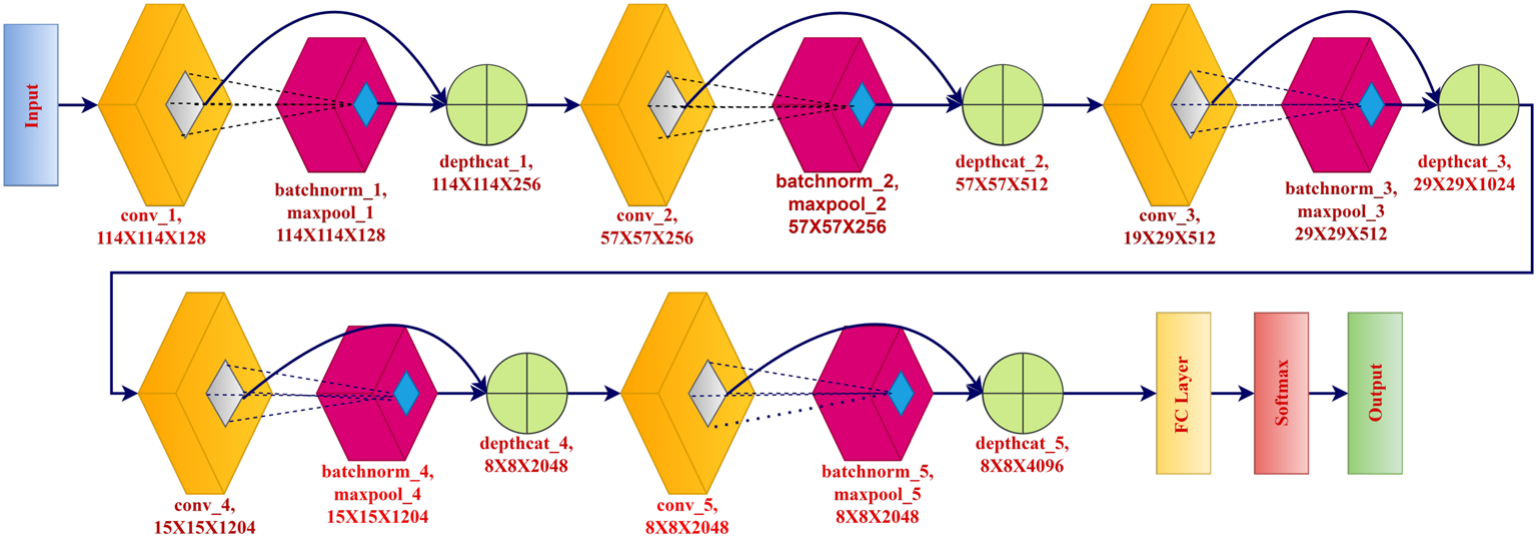
Architecture of CDNN.

This section describes the proposed model for classification of different white blood cells. Figure 2 shows the proposed model for classification of WBCs using CDNN. This classification of WBCs is done in three stages. **Stage 1**: In this, collection of datasets is taken place. Then dataset is undergo the image pre-processing, normalization, and data-augmentation. Then images are splitted into training and testing image datasets. Then augmentation is applied only for the training images. In this, images is resized to $227\times 227 \times 3$, and all are normalized to the pixel value [0 1]. In this we have used random rotation with value [-50 50], random reflection value 1, and random shear value [-5 5] augmentation techniques to avoid the over-fitting. **Stage 2**: In this, creating the DL model is done by defining the activations, filters, and kernels. In this, we have created the novel architecture to distinguish the WBCs known as CDNN. Then CDNN is trained with Adam optimizer^43^, 50 epochs, 64 batch size, initial learn rate 1e-5, learn rate drop rate 0.3. **Stage 3**: After training, classification of WBCs are taken place. In this, we have classified the Raabin WBCs into five classes and BCCD WBCs into four classes. The algorithm for the classification of the CDNN is presented in the Table. The structure of the CDNN is shown in the Fig. 3.

- **Step1:** Collection of datasets.
- **Step2:**] Splitting dataset into train and test.
- **Step3:** Data processing.
- **Step4:** designing CDNN model.
- **Step5:** Defining and tuning the CDNN training hyper-parameters.
- **Step6:** K-fold CV with K=5.^44^
- **Step7:** Classified output: four and five classes.
- **Step8:** Finding performance metrics.
- **Step9:** Applying G-CAM and LIME to images to understand the classification process

### CDNN(Leukocytes or white blood cells network)

This section describes the proposed novel Leukocytes or White Blood Cells Network (CDNN) architecture. The CDNN architecture and details of the layers used represented in the Fig. 3 and detailed layer parameters are listed in the Table 2. This architecture consists of four residual blocks and each residual blocks consist convolutional layer, ReLU activation layer, batch normalization layer, and maxpool layer with stride 2 and zero padding.

**Table 2:** Details of CDNN architecture.

Layer Name	Type	Activation’s	Learnables	Stride
Image Input	Image Input	227x227x3	-	
Conv1	Convolution	114x114x128	5x5x3x128 Bias 1x1x128	2,2
ReLU4	ReLU	114x114x128	-	
batchnorm1	Batch Normalization	114x114x128	Offset 1x1x128 Scale 1x1x128	
maxpool1	Max Pooliing	114x114x128	-	1,1
depthcat5	Depth Concatenation	114x114x256	-	
conv2	Convolution	57x57x256	Weights 5x5x256x256 Bias 1x1x256	2,2
ReLU3	ReLU	57x57x256	-	
batchnorm2	Batch Normalization	57x57x256	1x1x256 Scale 1x1x256	
maxpool2	Max Pooling	57x57x256	-	1,1
depthcat1	Depth Normalization	57x57x512	-	
conv3	Convolution	29x29x512	Weights 5x5x512x512 Bias 1x1x512	2,2
ReLU2	ReLU	29x29x512	-	
batchnorm3	Batch Normalization	29x29x512	Offset 1x1x512 Scale 1x1x512	
maxpool3	Max Pooling	29x29x512	-	1,1
depthcat2	Depth Concatenation	29x29x1024	-	
conv4	Convolution	15x15x1024	Weights 5x5x1024x1024 Bias 1x1x1024	2,2
ReLU1	ReLU	15x15x1024	-	
batchnorm4	Batch Normalization	15x15x1024	Offset 1x1x1024 Scale 1x1x1024	
maxpool4	Max Pooling	15x15x1024	-	1,1
depthcat3	Depth Concatenation	15x15x2048	-	
conv5	Convolution	8x8x2048	Weights 5x5x2048x2048 Bias 1x1x2048	2,2
ReLU5	ReLU	8x8x2048	-	
batchnorm5	Batch Normalization	8x8x2048	Offset 1x1x2048 Scale 1x1x2048	
maxpool5	Max Pooling	8x8x2048	-	1,1
depthcat4	Depth Concatenation	8x8x4096	-	
fc	Fully Connected	1x1x5	Weights 5x262144 Bias 5x1	
softmax	Softmax	1x1x5	-	
classoutput	Classification Output	1x1x5	-	

**Fig. 4:**
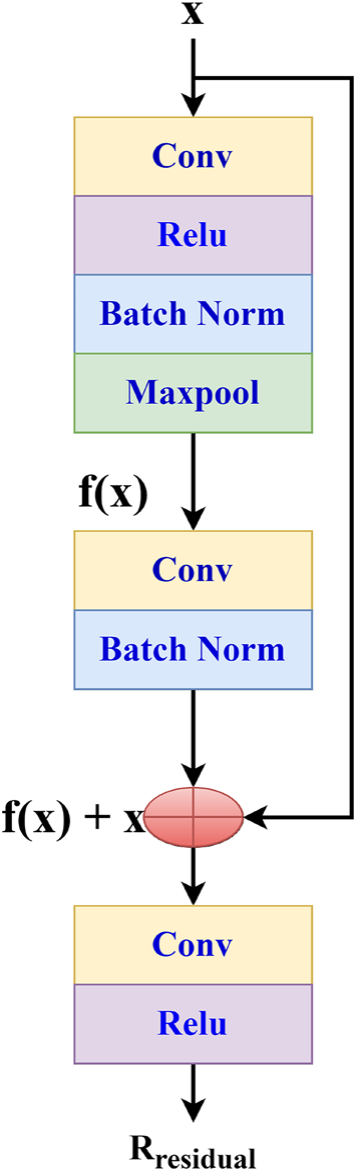
Residual block used in CDNN.

The partial derivatives’ components experience vanishing gradients as they approach exponentially tiny values relative to the neural network’s parameters, making the gradient’s effect on updating the parameters practically trivial. This is discernible by looking at the kernel weight distribution, and it is present when the weights frequently approach zero. When a neural network is trained very slowly, this issue might be found. With the data we are utilizing, neural networks are not properly trained, or the outputs exhibit strange behaviour. So, to prevent this problem we have used our novel proposed architecture CDNN that contains residual blocks and also we have used ReLU activation function. This residual block structure is shown in the Fig. 4.

This residual block output is represented as

1 $$\begin{aligned} R_{residual}=f(x, l_{wl})+ x \end{aligned}$$

where x is input and $R_{residual}$ is output vector; $l_{wl}$ weight of layer. If input and shortcut vectors are not matched we have to add convolutional layer with ReLU in shortcut path.This can be represented as

2 $$\begin{aligned} R_{residual} =f(x, l_{wl})+w_{s}x \end{aligned}$$

where $w_{s}$ is the weight of shortcut. The suggested CDNN is a concise and efficient architecture specifically developed for leukocyte categorization. Each of the four residual blocks comprises a convolutional layer utilizing a 5x5 kernel, batch normalization, max pooling, and ReLU activation. After each residual block, depth concatenation amalgamates features from different scales in the prior layers. This guarantees the conservation of both macro- and micro-morphological characteristics. This method enables the network to maintain more extensive feature representations than conventional sequential CNNs. Training progresses consistently as the model deepens, facilitated by residual learning’s capacity to mitigate disappearing gradients. The ReLU activation function guarantees a non-linear transformation of features. The architecture initiates with 128 filters in the initial convolution and culminates with 2048 in the final convolution, processing images of dimensions $227 \times 227 \times 3$ pixels while incrementally increasing the number of filters. A fully connected layer including five output neurons is linked to the final output, which is depth-concatenated. The data is subsequently categorized utilizing a softmax activation function. The CDNN is efficient and user-friendly in resource-constrained applications, necessitating merely 10 MB of memory and comprising 1,310,720 trainable parameters. CDNN is significantly smaller than conventional CNN backbones, yet it performs exceptionally well in the leukocyte categorization challenge. For instance, VGG16 comprises 138 million parameters with 528MB of memory size, ResNet-50 possesses 25.6 million parameters and need 98MB of memory, and DenseNet-121 includes 8 million parameters required about 33MB of memory size. Conversely, CDNN possesses merely 1.31 million parameters and occupies 10 MB of storage. This renders it over 10 times smaller than DenseNet-121 and over 50 times smaller than VGG16. The specific architecture of the residual block and the depth concatenation technique enable a substantial alteration in size without compromising classification performance.

Before classification of WBCs, model must first be trained using a set of parameters. Because it can improve network weights based on training data, Adam optimizer is used to train the model with 50 epochs, 64 batch size and K=5 cross validation for the classification. After training, the model is tested using test set data. The setting up of K=5 graded cross-validation guaranteed robustness and reliability. K=10 may enhance the consistency of performance estimations; however, it would need double the training time without augmenting classification stability. This decision created an equitable equilibrium between expense and statistical dependability. In medical imaging tasks where class imbalance may affect learning, the stratified approach guaranteed that each fold preserved the baseline class distribution. To achieve an impartial evaluation of the WBC classification model’s performance, it was retrained on the complete training dataset and subsequently assessed on a separate held-out test set after cross-validation.

## Experimental results

**Fig. 5:**
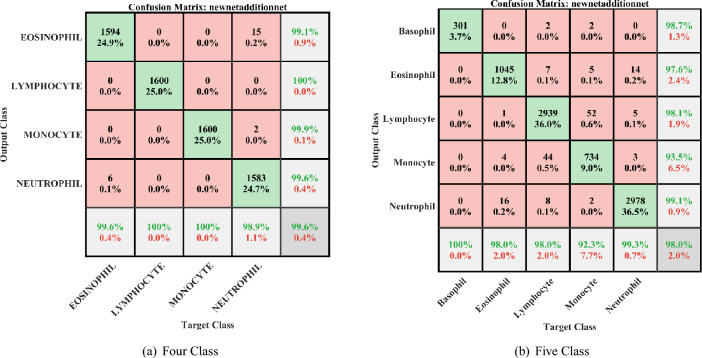
Confusion matrices of CDNN for WBCs classification.

**Fig. 6:**
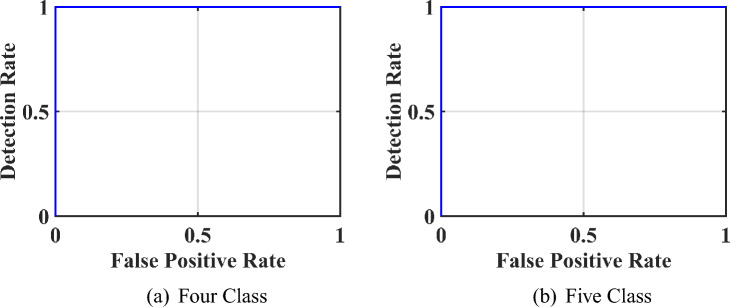
ROCs of CDNN for WBCs classification.

This work is implemented in Matlab2021a in 32GB RAM and 1TB SSD desktop. We used Raabin and BCCD two datasets to check the performance of the proposed novel architecture. In these, Raabin is imbalanced and BCCD is uniform and balanced dataset. Raabin dataset has five classes of WBCs and BCCD dataset has four classes. In this, we have calculated eight performance measures such as area under curve (AUC), accuracy, sensitivity, specificity, precision, recall, f-score, and geometric mean(G-mean) from the confusion matrices(CM) shown in the Fig. 5 and region of convergence (ROC) of BCCD and Raabin dataset classification shown in the Fig. 6. Figure 5a shows the CM of BCCD dataset of four-class classification. In this, WBCs are classified into four classes as eosinophil, lymphocyte, monocyte, and neutrophil. Eosinophil is classified with an accuracy of 99.1%, lymphocyte is classified with 100% accuracy, monocyte is classified with 99.9%, neutrophil is classified with 99.6% of accuracy and overall average accuracy of 99.64%. Figure 5b shows the CM of Raabin dataset classification that has five-class classification. In this, basophil is classified with 98.7%, eosinophil is classified with 97.6%, lymphocyte is classified with 98.1%, monocyte is classified with 93.5%, neutrophil is classified with 99.1%, and overall average accuracy of 98% with overall loss of 2%. Using these CMs, the performance matrices of the network are tabulated in the Table 3.

Table 3 shows the performance analysis of the CDNN. From Table Raabin dataset has the classification accuracy of 97.97%, sensitivity or recall of 98.03%, specificity of 97.97%. precision of 87.88%, f1-score of 92.68%, and G-mean of 98.00%. For BCCD dataset has the classification accuracy of 99.64%, sensitivity or recall of 99.06%, specificity of 99.64%, precision of 99.62%, f1-score of 99.34%, and g-mean of 99.03%. From these, classification of BCCD datasets achieved highest accuracy.

**Table 3:** Performance evaluation metrics of two datasets with K=5.

Performance metrics	Raabin dataset	BCCD
AUC	0.9998	0.9999
Accuracy	97.97	99.64
Sensitivity	98.03	99.06
Specificity	97.97	99.64
Precision	87.88	99.62
Recall	98.03	99.06
F1-Score	92.68	99.34
G-Mean	98.00	99.03

**Table 4:** Comparison of the proposed model with DL-models for BCCD dataset.

Method	Accuracy	Sensitivity	F1-score	Precision
GoogleNet	91.00	87.00	88.00	88.00
SqueezeNet	96.00	97.00	96.00	97.00
AlexNet	87.00	86.00	86.00	86.00
EfficientNetV2	90.00	89.00	89.00	89.00
Xception	82.00	81.00	82.00	82.00
VGG19	95.00	96.00	96.00	94.00
ResNet50	98.00	89.00	89.00	89.00
LSTM	97.00	97.00	97.00	97.00
**Proposed**	**99.64**	**99.06**	**99.34**	**99.62**

Table 4 shows the comparison of the model with different DL- models on BCCD dataset. From Table 4, it is observed that the proposed model outperformed the conventional DL-models^45,46^. Also, proposed model has trained with less parameters and required the less memory compared to the other DL- models listed in the Table 4.

Critical considerations for implementation in resource-constrained environments within the proposed CDNN architecture for leukocyte classification are computational complexity, training duration, and memory consumption. The method uses five convolutional layers and multiple depth concatenation operations, which greatly increases the number of learnable parameters and the computing power needed for both the forward and backward processes. The model, comprising around 1,310,720 trainable parameters, necessitates around 10 MB of memory during training. While this is relatively modest compared to more sophisticated models such as ResNet or VGG, real-time applications still require moderate GPU or high-performance CPU resources. Every convolutional layer employs $5\times 5$ filters, which are computationally more demanding than the smaller $3\times 3$ kernels often employed in lightweight architectures. Furthermore, following each residual block, depth concatenation doubles the dimensions of the feature map, hence increasing both memory and computing expenses. Training was conducted over 50 epochs with a batch size of 64, the Adam optimizer, and 5-fold cross-validation. Consequently, the CDNN model attains a balance between accuracy and parameter size, further efforts to reduce computational burden are essential for use in low-power environments such as mobile health applications or embedded diagnostic devices.

## Discussion

The WBCs is an important task in clinical pathology as it helps in the diagnosis of various diseases. Traditionally, the classification of WBCs has been performed manually that is a time-consuming and error-prone process. However, with the advent of models, automatic classification of WBCs has become possible.

### Visualization of WBCs using Gradient Activation Map(GCAM) and local interpretable model-agnostic explanations (LIME)

**Fig. 7:**
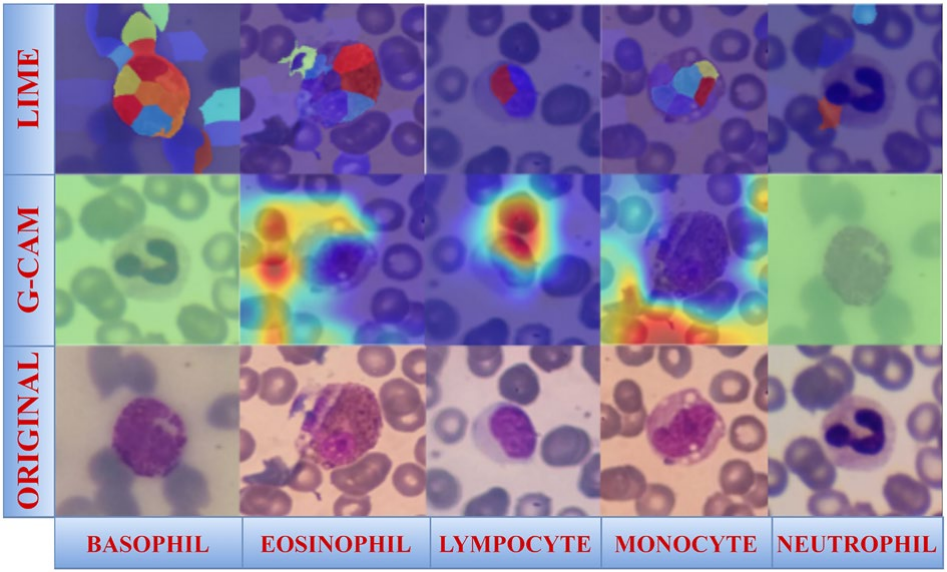
Visualization using LIME and G-CAM.

G-CAM and Lime are used once the projected label has been produced using the complete model. Figure 7 displays the G-CAM on the input pictures. GCAM utilized the jet color scheme to illustrate the contribution of different image components to the prediction. The areas where the network predominantly emphasizes class-specific properties are denoted by red and dark red regions in these heatmaps. The network exhibits partial emphasis on class-specific characteristics, as denoted by yellow and green regions. The model fails to extract any class-relevant features in the blue regions. These patterns illustrate that the model emphasizes morphologically significant cellular components, including the cytoplasm and nucleus. LIME visualization segment the image into super-pixels and emphasize the areas that most significantly impact the classification decision. The blue regions in the LIME overlays signify areas that provide little assistance, whereas brown, orange, and other warm hues denote areas that support the anticipated class. These visual indicators facilitate the classifier’s ability to produce predictions grounded in physiologically relevant features rather than extraneous background patterns. We presented class-discriminative localization (G-CAM) and feature importance (LIME) at the pixel level using both G-CAM and LIME. Examining the annotated instances in Fig. 7 may enhance our comprehension of the CDNN’s decision-making process and confirm that its predictions are substantiated by medically pertinent evidence.

### Network Behavioral study using t-SNE

**Fig. 8:**
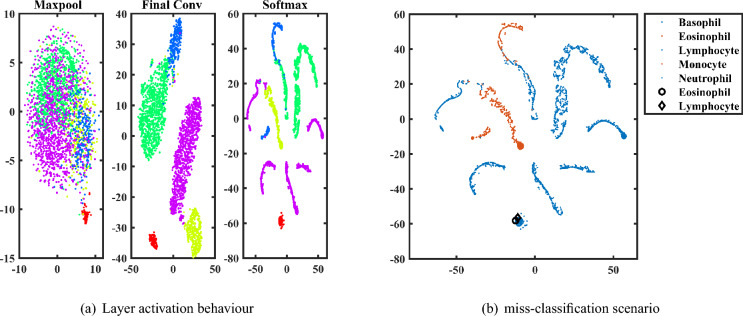
CDNN behaviour using t-SNE plot.

The behaviour of the network is shown in Fig. 8 at maxpool activation (MPA), final convolutional activation (FCA), and softmax layer activation (SFA) after the Distributed Stochastic Neighbor Embedding algorithm has been applied (t-SNE). The behaviour of the network is seen in Fig. 8a for five different class classifications. Two clusters are shown here, with four distinct clusters accounting for the four different classes. As compared to the other layers, the scattering plot of the SFA layer is more distinct and it has a higher degree of accuracy. This suggests that activations of deeper layers provide more accurate results when used to the categorization of images. The dispersion plot Fig. 8b that was created with the t-SNE approach provides an instance of miss-classification characteristics for five class classification. In this, forms like diamonds and rounded shapes signify classes that have been misclassified and are seen inside other class clusters. As a consequence of this, the scatter plots help in identifying the problems associated with the network’s misclassification.

### Miss-classification analysis

**Fig. 9:**
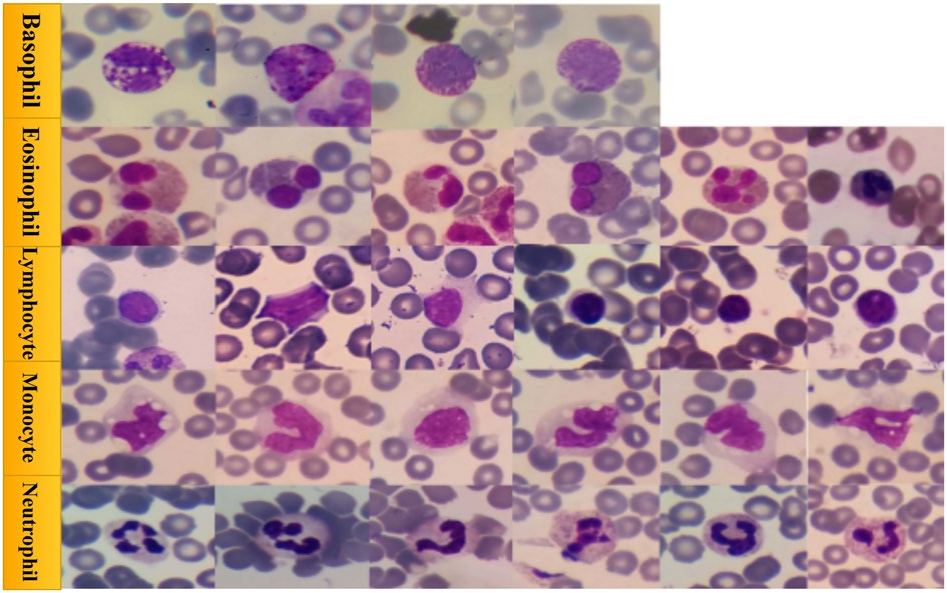
Common miss-classified images.

Table 5 represents the miss-classified images with respect to Raabin and BCCD datasets. Totally 166 images are miss-classified from Raabin dataset and 17 images are miss-classified from BCCD datasets. The common miss-classified images of each class is shown in the Fig. 9. This miss-classification can be further studied in future. This can be reduced by applying different image pre-processing techniques, optimization, and hyper-parameter tuning.

**Table 5:** Number of miss classified images from each datasets with respect to type of blood cell.

Raabin dataset	MCI	BCCD dataset	MCI
Basophil	4	—	
Eosinophil	26	Eosinophil	15
Lymphocyte	58	Lymphocyte	0
Monocyte	51	Monocyte	2
Neutrophil	26	Neutrophil	6

*MCI: Miss classified images.

### Comparison with existing state-of-the-art methods

Models such as CNNs, recurrent neural networks (RNNs), and hybrid models have been used for WBC classification. These models have shown promising results on various datasets, including publicly available datasets such as the BCCD and the Peripheral Blood Cell Dataset (PBC). The use of models has resulted in high accuracy and efficiency in WBC classification, which can lead to improved diagnosis and treatment of diseases.

Table 6 represents the comparison of proposed model with state-of-the-art models. In^47–50^ used basic CNNs for the classification of WBCs and achieved an accuracy of 96.8%, 98.9%, 97.9%, and 97.8% respectively.^51^ used the capsule network for the classification of the WBCs that achieved an accuracy of 96.9%. Deep CNN (DCCN) is used for the classification of WBCs in^52,53^ with an accuracy of 98.9% and 98.8%. Fusion of AlexNet, GoogleNet, and SVM was used in the classification of WBCs from BCCD dataset with an highest accuracy of 99.7% in^11^.^54^ proposed the transfer learning CNN for the classification of WBCs with an accuracy of 98.7%. In^55^ used modulated CNN for the classification of the WBCs and achieved the an accuracy of 97.7%.^56^ proposed CDNN to classify the WBCs and achieved an accuracy of 83%. In^57^ used the multilayer AlexNet for the classification and achieved an accuracy of 99.1% where as in^7^ used AlexNet and achieved an accuracy of 95.9%. RCNN is used for the classification of CDNN in^58^ and in^59^ used RNN+CNN is used with an accuracy of 97% and 96.03% respectively. In^12,50,60^ used fusion CNN, MC-CNN and basic CNN are used for CDNN classification with an accuracy of 97.7%, 90.5%, and 98.4% respectively.^3^ proposed canonical correlation analysis for the four class classification of WBCs and achieved the accuracy of 98.4%. Finally, observing all these model, our proposed CDNN achieved the highest accuracy of 99.64%, sensitivity of 99.06% and 99.34% of f1-score by preventing over-fitting, gradient vanishing using ReLU and residual blocks in the network.

**Table 6:** Comparison of proposed method with previous state-of-the-art methods.

Model	Method	Accuracy (%)	Sensitivity (%)	F1 Score (%)
^47^	CNN	96.80	NA	NA
^48^	CNN	98.90	97.70	97.60
^51^	Capsule Network	96.90	92.50	92.30
^49^	CNN	97.90	98.60	97.00
^52^	DCNN	98.90	97.80	97.70
^11^	AlexNet+GoogleNet+SVM	99.70	99.00	99.00
^54^	TL-CNN	98.70	99.00	99.00
^55^	Modulated Gabor CNN	97.70	NA	NA
^56^	CDNN	83.00	NA	NA
^57^	Multilayer AlexNet	99.10	99.00	99.00
^58^	RCNN	97.00	99.00	98.00
^61^	DCNN and Extreme Machine Learning	95.40	96.90	94.00
^59^	RNN+CNN	96.03	NA	NA
^7^	AlexNet	95.90	95.80	95.80
^53^	3DCNN	98.80	95.90	96.40
^50^	CNN	97.80	95.70	95.60
^60^	Fusion CNN	97.70	NA	NA
^3^	Canonical Correlation Analysis	95.70	95.70	95.70
^62^	Weighted optimized deformable CNN	90.50	92.40	86.60
^12^	MC-CNN	98.40	98.40	98.40
**Proposed CDNN**	**CDNN**	**99.64**	**99.06**	**99.34**

The proposed CDNN model offers numerous advantages for the classification of WBCs. Despite possessing about 1.31 million parameters, it remains lightweight and exhibits strong performance on the Raabin and BCCD datasets, indicating effective generalization across diverse data sources. The consistent application of $5\times 5$ kernels facilitates the extraction of characteristics from the cytoplasm and nucleus. Depth concatenation and residual learning mitigate gradient vanishing and enhance feature representation. The model’s compact size and minimal computational requirements enable its deployment on edge or low-resource devices. The use of Grad-CAM and LIME visualizations enhances understanding of the model and illustrates that the predictions are based in physiologically relevant areas. Nonetheless, the model possesses numerous deficiencies as well. Alterations in staining techniques or imaging settings not seen during training may affect its performance. Although batch normalization and residual connections mitigate overfitting, the lack of dropout regularization may diminish the models’ robustness in the presence of severely unbalanced or noisy data. The model presently lacks components for assessing prediction uncertainty, which could be advantageous in clinical applications, and the examination of misclassified instances is constrained. Ultimately, while it demonstrates proficiency in classifying WBCs, its architecture may require modification to perform effectively in alternative medical imaging applications.

## Conclusion

a model called CDNN is designed to categorise various WBCs. The normalization and DA preparation approaches optimize the CDNN model. The Raabin dataset and the BCCD dataset were both used in this study as WBC datasets. When compared to other cutting-edge models and pre-trained models, the suggested model, which is trained with the Adam optimizer and ran over 50 epochs with batch size 32 of CDNN, produces the best results. The suggested novel CDNN model attained accuracy, precision, sensitivity, and specificity values of 99.64%, 99.62%, 99.06%, and 99.64% for BCCD dataset. For Raabin datasets this model achived an accuracy of 97.97%, sensitivity of 98.03%, precision of 87.88%, and specificity of 97.97% . Based on the findings, it can be said that this model outperforms alternative lot sizes in the BCCD dataset with BS 32. This novel CDNN model stays clear of issues including degradation, gradient vanishing, and over-fitting. Finally, we applied the visualization techniques to understand the area that is used more for the classification and differentiate the different WBC classes. The early detection of WBC is the major objective of this study. This comparative analysis methodology may be used by pathologists as a second opinion tool. Our findings suggest that these models might be utilised to create therapeutically beneficial WBC detection tools for blood cell images.

## Data Availability

First Author will share the Data based on the request and scratch code available in provided link: https://www.kaggle.com/datasets/sahebgoudkaraddi/cdnn-for-wbc-classification.
